# Reduced kidney function is associated with poorer domain‐specific cognitive performance in community‐dwelling older adults

**DOI:** 10.1002/gps.5771

**Published:** 2022-06-19

**Authors:** Adam H. Dyer, Eamon Laird, Leane Hoey, Catherine F. Hughes, Helene McNulty, Mary Ward, J. J. Strain, Maurice O’Kane, Fergal Tracey, Anne M. Molloy, Conal Cunningham, Donal J. Sexton, Kevin McCarroll

**Affiliations:** ^1^ Mercer's Institute for Successful Ageing St James's Hospital Dublin Ireland; ^2^ Wellcome‐HRB Clinical Research Facility St James's Hospital Dublin Ireland; ^3^ Department of Medical Gerontology School of Medicine Trinity College Dublin Dublin Ireland; ^4^ The Nutrition Innovation Centre for Food and Health (NICHE) School of Biomedical Sciences Ulster University Coleraine Northern Ireland UK; ^5^ Clinical Chemistry Laboratory Altnagelvin Hospital Western Health and Social Care Trust Londonderry Northern Ireland UK; ^6^ Causeway Hospital Northern Health and Social Care Trust Coleraine Northern Ireland UK; ^7^ School of Medicine Trinity College Dublin Dublin Ireland; ^8^ Trinity Health Kidney Centre School of Medicine Trinity College Dublin Dublin Ireland

**Keywords:** chronic kidney disease (CKD), cognition, older adults

## Abstract

**Objectives:**

Whilst chronic kidney disease has been associated with cognitive impairment, the association between reduced estimated Glomerular Filtration Rate (eGFR) and domain‐specific cognitive performance is less clear and may represent an important target for the promotion of optimal brain health in older adults.

**Methods:**

Participants aged >60 years from the Trinity‐Ulster‐Department of Agriculture study underwent detailed cognitive assessment using the Mini‐Mental State Examination (Mini‐Mental State Examination (MMSE)), Frontal Assessment Battery (FAB) and Repeatable Battery for Assessment of Neuropsychological Status (RBANS). Poisson and linear regression models assessed the relationship between eGFR strata and cognitive performance.

**Results:**

In 4887 older adults (73.9 ± 8.3 years; 67.7% female), declining eGFR strata was associated with greater likelihood of error on the MMSE/FAB and poorer overall performance on the RBANS. Following robust covariate adjustment, findings were greatest for GFR <45 ml/ml/1.73 m^2^ (Incidence Rate Ratio: 1.17; 95% CI 1.08, 1.27; *p* < 0.001 for MMSE; IRR: 1.13; 95% CI 1.04, 1.13; *p* < 0.001 for FAB; β: −3.66; 95% CI −5.64, −1.86; *p* < 0.001 for RBANS). Additionally, eGFR <45 ml/ml/1.73 m^2^ was associated with poorer performance on all five RBANS domains, with greatest effect sizes for immediate memory, delayed memory and attention. Associations were strongest in those aged 60–70, with no associations observed in those >80 years.

**Conclusions:**

Reduced kidney function was associated with poorer global and domain‐specific neuropsychological performance. Associations were strongest with eGFR <45 ml/min/1.73 m^2^ and in those aged 60–70 years, suggesting that this population may potentially benefit from potential multi‐domain interventions aimed at promoting optimal brain health in older adults.

## Key points

- Reduced kidney function was associated with poorer performance on tests of global and domain‐specific cognitive function in older adults.
- In particular, reduced kidney function in older adults was associated with poorer performance in tests of immediate memory, delayed memory and attention.
- Associations were strongest in those aged 60–70 years and in those with estimated Glomerular Filtration Rate (eGFR) <45 ml/min/1.73 m^2^.

## 1 INTRODUCTION

Chronic Kidney Disease (CKD) and dementia disproportionately affect older adults.^1^ Further, evidence has accumulated that reduced kidney function, assessed using eGFR, is associated with a greater risk of incident cognitive impairment and dementia in older adults.^2^, ^3^ Whilst this association has been supported by several well‐conducted cohort studies and meta‐analyses,^2^, ^3^, ^4^, ^5^, ^6^, ^7^, ^8^, ^9^, ^10^, ^11^, ^12^ fewer studies have examined the detailed relationship between impaired kidney function and detailed domain‐specific neuropsychological performance.

Identifying which cognitive and neuropsychological domains are first affected in older adults with CKD is important in selecting‐out those at greatest risk of potential cognitive decline, who may benefit from preventative interventions which have proven efficacy in delaying cognitive decline in high‐risk individuals.^13^ Whilst previous studies have largely examined the impact of CKD on incident cognitive impairment or dementia, few studies have examined the influence of CKD on more subtle cognitive decrements in domain‐specific neuropsychological performance, which may represent targets for multi‐domain preventative interventions in the future.

In one of the earliest reports examining the CKD‐dementia link (Cardiovascular Health Cognition Study), older adults with CKD experienced a 37% increased risk of dementia at 6‐year follow‐up.^4^ Similarly, in the Health, Aging and Body composition study, older adults with CKD experienced a greater likelihood of cognitive decline at 2‐ and 4‐year follow‐up.^5^ Similar results from other studies soon followed.^6^, ^7^, ^8^, ^9^, ^10^, ^11^, ^12^, ^13^, ^14^, ^15^, ^16^ A meta‐analysis (n > 50,000 individuals) estimated that reduced kidney function (eGFR <60 ml/min/1.73 m^2^) is associated with a 65% increased risk of incident cognitive decline.^2^ Most studies have used a limited number of cognitive tests, most typically the Mini‐Mental State Examination (MMSE) which lacks the sensitivity to detect significant relationships with domain‐specific cognitive function.^2^ Furthermore, the association between reduced eGFR and cognitive performance using detailed neuropsychological assessment batteries has been less well‐explored in large studies.

Of note, early‐stage CKD has been associated with a drop in processing and response speed, attention and short‐term memory, whilst moderate CKD was significantly associated with deficits in executive functioning, verbal fluency, logical memory, orientation and concentration.^17^ In a noteworthy study of 898 individuals assessed using 22 measures of cognitive ability, reduced eGFR (<60 ml/min/1.73 m^2^) was associated with a greater risk of impairment in visuo‐spatial organisation/memory domains in addition to impairments in language, scanning and tracking.^18^ Similar findings have been reported for verbal learning, visual memory and frontal‐executive function.^19^, ^20^, ^21^ Whilst such studies have begun to characterise the patterns of cognitive impairment seen in individuals with CKD, a more in‐depth investigation of the relationship between reduced eGFR and domain‐specific cognitive and neuropsychological performance is warranted.

The Trinity‐Ulster and Department of Agriculture (Trinity‐Ulster‐Department of Agriculture (TUDA)) study enrolled over 5000 community‐dwelling older Irish adults, free from an established diagnosis of dementia. Uniquely, this study employed a battery of cognitive and neuropsychological tests evaluating global cognition, executive function and domain specific neuropsychological performance. A primary aim of the TUDA study was to examine predictors of cognitive performance in older adults, offering a unique opportunity to interrogate the relationship between reduced eGFR and cognitive dysfunction over a multitude of cognitive domains. In the current study, we examined the relationship between declining kidney function (in eGFR strata) and performance on a detailed neuropsychological battery to examine the relationship between reduced kidney function and subtle neuropsychological impairments in community‐dwelling older adults.

## 2 METHODS

### 2.1 Study setting and participants

The current study analysed data from the TUDA study (ClinicalTrials.gov identifier: NCT02664584). Ethical approval was granted from the Office for Research Ethics Committees Northern Ireland (ref: 08/NIR03/113), and the Research Ethics Committee in St James's Hospital, Dublin, Ireland. Adults aged 60 years and older, free from a diagnosis of dementia, were recruited as part of three pre‐specified sub‐cohorts: (i) cognitive: from geriatric medicine clinics/day hospital, (ii) bone: from a specialist bone health service and (iii) hypertensive: individuals with hypertension recruited from general practices. Reports of the TUDA study methodology have been published elsewhere.^22^, ^23^, ^24^, ^25^

### 2.2 Health assessment and clinical covariates

Participants attended for a 90‐min interview. Weight and height were measured in a standardized fashion. Blood pressure was measured in the seated position using a 705 CP‐II blood pressure monitor (Omron, Milton Keynes, UK.). Participants were asked to self‐report specific medical comorbidities (hypertension, myocardial infarction, atrial fibrillation, angina, ischaemic heart disease, diabetes, previous stroke, previous Transient Ischaemic Attack, TIA) and medication usage (coded using the Anatomic Therapeutic Classification: ATC system). Alcohol and smoking status were obtained by self‐report (never/former/current).

### 2.3 Blood sampling and analysis

Fasting blood samples were processed within 4 h of collection. Total cholesterol and high density lipoprotein were analysed in hospital laboratories in a standardised fashion. Creatinine was also measured in the hospital laboratory and reported in μmol/L. The Clinical Kidney Disease Epidemiology Collaboration (CKD‐Epi) formula was applied for eGFR as used in previous studies examining associations between eGFR and outcomes in community‐dwelling older adults.^26^, ^27^, ^28^ eGFR was divided into strata as follows: (i) ≥ 90 ml/min/1.73 m^2^ (reference range); (ii) 75–89.9 ml/min/1.73 m^2^; (iii) 60–74.9 ml/min/1.73 m^2^, (iv) 45.0–59.9 ml/min/1.73 m^2^ and <45 ml/min/1.73 m^2^, consistent with previous studies.^27^, ^28^

### 2.4 Cognitive and neuropsychological assessment

Participants underwent detailed cognitive and neuropsychological assessment as part of the TUDA study. General cognition was screened using the MMSE.^29^ The Frontal Assessment Battery (FAB) was used as a test of executive function and includes domains of conceptualization (assessing similarities), mental flexibility (verbal fluency), motor programming (‘Luria’ test), resistance to interference (conflicting instructions), inhibitory control (via a go‐no go paradigm) and environmental autonomy (behaviour).^30^ Finally, RBANS was used as a comprehensive neuropsychological assessment battery of immediate memory (Index I), visuo‐spatial (Index II), language (Index III), attention (Index IV) and delayed memory (Index V) domains.^31^

### 2.5 Statistical analysis

All data were analysed in STATA v15.1. Descriptive statistics were generated as means (with standard deviations), medians (with interquartile ranges) and proportions (with percentages) as appropriate. Between group differences were assessed by renal function strata using ANOVA, Kruskal‐wallis and Chi‐square tests. For analysing the association between eGFR strata and cognitive function, mixed‐effects models were used with random effects for study site to account for potential site‐specific effects.

Given the skew of both MMSE and FAB scores, we analysed the likelihood of error on these measures using a Poisson regression model (and confirmed Poisson was a better model fit in comparison to a negative binomial model to check for over‐dispersion). RBANS scores were normally distributed and mixed‐effects linear regression was used to analyse the association between eGFR strata and cognitive function on the RBANS total as well as specific RBANS domains. For linear models, variance inflation factors were calculated and residual versus fit plots examined post‐hoc.

In the first instance, we performed the analysis unadjusted (Model 1). In the next model, we adjusted for age, sex, body mass index and educational level (Model 2). Model 3 adjusted for all covariates from model 2 in addition to systolic blood pressure and diastolic blood pressure, history of diabetes, total cholesterol:HDL ratio, cardiovascular disease (a count of 0, 1, 2+of ischemic heart disease, atrial fibrillation, previous MI, history of angina, percutaneous coronary intervention or coronary artery bypass graft or congestive cardiac failure), cerebrovascular disease (previous TIA or stroke), polypharmacy (5 or more regular medications), regular use of an Angiotensin‐Converting Enzyme Inhibitor or Angiotensin Receptor Blocker , alcohol (none/former/current) and smoking (none/former/current) status.

In secondary analysis, we examined potential associations between eGFR strata and cognitive dysfunction within age strata. This analysis was performed to examine for any age‐specific associations. Age was classified as follows: (i) < 70 years, (ii) 70–80 years and (iii) > 80 years. We re‐ran all of the models within each age strata to examine for age‐specific associations.

For Poisson models, results are presented as Incidence Rate Ratios with corresponding 95% Confidence Intervals (95% CI) and for linear models results are presented as beta coefficients with corresponding 95% CI.

## 3 RESULTS

Of 5186 participants recruited into the TUDA study, 4887 (age: 73.94 +/− 8.25; 67.7% female) had full data available for inclusion in the current analysis. Overall, 1401 (28.7%) participants had an eGFR of 75.0–89.9 ml/min/1.73 m^2^, 1398 (28.4%) had an eGFR of 60–74.9 ml/min/1.73 m^2^, whilst 956 (19.6%) had an eGFR of 45.0–59.9 ml/min/1.73 m^2^. A minority, 562 (11.50%) had an eGFR <45.0 ml/min/1.73 m^2^. Baseline characteristics by eGFR strata are provided in Table 1. As expected, age and the presence of cardiovascular risk factors and conditions differed significantly by eGFR strata, necessitating inclusion of these covariates in subsequent multivariate models.

**TABLE 1:** General characteristics of Trinity‐Ulster‐Department of Agriculture (TUDA) participants by kidney function

Characteristic	eGFR_creat_ >90 ml/min (n = 579)	eGFR_creat_ 75.0–89.9 ml/min (n = 1401)	eGFR_creat_ 60.0–74.9 ml/min (n = 1389)	eGFR_creat_ 45.0–59.9 ml/min (n = 956)	eGFR_creat_ <45 ml/min (n = 562)	Statistic
Age, years (SD)	66.5 (4.6)	72.7 (7.3)	73.5 (7.9)	76.8 (8.1)	80.84 (7.3)	*F* = 316, *p* < 0.001
Sex, female n (%)	405 (70.0%)	925 (66.0%)	916 (66.0%)	664 (69.5%)	399 (71.0%)	χ^2^ = 9.3, *p* = 0.06
Body Mass index (SD)	26.6 (5.6)	27.6 (5.2)	28.4 (5.4)	28.43 (5.3)	27.81 (5.6)	*F* = 16, *p* < 0.001
Education, age finished (SD)	16.5 (3.3)	16.3 (3.1)	16.1 (2.9)	15.8 (2.6)	15.75 (3.2)	*F* = 8.5, *p* < 0.001
Hypertension history n (%)	316 (54.6%)	997 (71.2%)	1094 (78.8%)	799 (83.6%)	486 (86.5%)	*F* = 229.9, *p* < 0.001
Diabetes history n (%)	38 (6.6%)	149 (10.6%)	170 (12.2%)	143 (15.0%)	109 (19.4%)	χ^2^ = 53.05, *p* < 0.001
Stroke/TIA history n (%)	49 (8.5%)	172 (12.3%)	176 (12.7%)	163 (17.1%)	144 (25.6%)	χ^2^ = 87.73, *p* < 0.001
Atrial fibrillation n (%)	38 (6.6%)	134 (9.6%)	154 (11.1%)	162 (17.0%)	142 (25.3%)	χ^2^ = 129.5, *p* < 0.001
Ischaemic heart disease n (%)	25 (4.3%)	164 (11.7%)	204 (14.7%)	199 (20.8%)	169 (30.1%)	χ^2^ = 182.9, *p* < 0.001
Polypharmacy n (%)	291 (56.5%)	814 (58.1%)	886 (63.8%)	721 (75.4%)	503 (89.5%)	χ^2^ = 274.8, *p* < 0.001
ACEI/ARB use	185 (32.0%)	577 (41.2%)	689 (49.6%)	531 (55.5%)	350 (62.3%)	χ^2^ = 154.9, *p* < 0.001
SBP (SD), mmHg	140.93 (19.7)	145.82 (20.4)	145.19 (20.6)	144.68 (21.6)	143.53 (23.9)	*F* = 6.2, *p* = 0.001
DBP (SD), mmHg	79.96 (10.7)	79.47 (10.7)	79.24 (11.2)	76.81 (11.3)	74.33 (11.9)	*F* = 31.8, *p* < 0.001
Total cholesterol (SD) in mmol/L	4.92 (1.0)	4.69 (1.0)	4.68 (1.1)	4.52 (1.0)	4.28 (1.0)	*F* = 32.1, *p* < 0.001
High density lipoprotein (SD) in mmol/L	1.65 (0.6)	1.53 (0.5)	1.46 (0.4)	1.42 (0.4)	1.36 (0.5)	*F* = 37.1, *p* < 0.001
Smoking						
Never n (%)	223 (38.5%)	652 (46.5%)	679 (48.8%)	468 (49.0%)	267 (47.5%)	
Former n (%)	235 (40.6%)	578 (41.3%)	567 (40.8%)	385 (40.3%)	250 (44.5%)	
Current n (%)	121 (20.9%)	171 (12.2%)	143 (10.3%)	103 (10.8%)	45 (8.0%)	χ^2^ = 63.1, *p* < 0.001
Alcohol						
Never n (%)	82 (14.2%)	292 (20.9%)	361 (26.0%)	293 (30.7%)	187 (33.3%)	
Former n (%)	76 (13.2%)	226 (16.1%)	238 (17.1%)	196 (20.5%)	130 (23.1%)	
Current n (%)	420 (72.7%)	883 (63.0%)	790 (56.9%)	466 (48.8%)	245 (43.6%)	χ^2^ = 150.1, *p* < 0.001
Mini‐mental state examination score, median (IQR)	28 (27–29)	28 (26–29)	28 (26–29)	27 (26–28)	27 (25–28)	χ^2^ = 201
						*p* < 0.001
Frontal assessment battery score, median (IQR)	17 (15–18)	16 (15–17)	16 (14–17)	16 (14–17)	15 (12–17)	χ^2^ = 188
						*p* < 0.001
Total RBANS score, median (IQR)	91 (80–103)	87 (77–100)	86 (77–98)	83 (72–94)	78 (66–89)	χ^2^ = 216
						*p* < 0.001
RBANS immediate memory score, median (IQR)	97 (85–109)	90 (81–103)	90 (98–103)	87 (76–100)	85 (73–97)	χ^2^ = 127
						*p* < 0.001
RBANS visual‐spatial score, median (IQR)	92 (82–109)	89 (75–105)	89 (75–102)	87 (72–100)	81 (64–92)	χ^2^ = 140
						*p* < 0.001
RBANS language score, median (IQR)	92 (88–99)	92 (86–99)	92 (85–98)	92 (85–97)	89 (79–96)	χ^2^ = 101
						*p* < 0.001
RBANS attention score, median (IQR)	91 (82–106)	91 (79–103)	88 (79–103)	85 (75–97)	82 (70–91)	χ^2^ = 175
						*p* < 0.001
RBANS delayed memory score, median (IQR)	98 (84–102)	93 (78–101)	91 (75–101)	86 (71–98)	84 (66–96)	χ^2^ = 150
						*p* < 0.001

*Note*: Data are presented as mean (standard deviations) and proportions (percentages) as indicated. Statistical analysis was performed using ANOVA and Kruskal‐Wallis tests as appropriate. Cognitive Tests are Presented as Medians with Interquartile Ranges.

Abbreviations: ACEI, Angiotensin‐Converting Enzyme Inhibitor; ARB, Angiotensin Receptor Blocker; DBP, Diastolic Blood Pressure; eGFR, Estimated Glomerular Filtration Rate; SBP, Systolic Blood Pressure; TIA, Transient Ischaemic Attack; TUDA, Trinity Ulster Department of Agriculture Study.

In unadjusted models, decreasing eGFR strata was associated with poorer performance on all three cognitive tests. The association between decreasing eGFR and likelihood of error on the MMSE persisted following robust covariate adjustment and was most pronounced for eGFR <45 ml/min/1.73 m^2^ (IRR: 1.17; 95% CI: 1.08, 1.27; *p* < 0.001). For the FAB and total RBANS scores, only the lowest strata of eGFR (<45 ml/min/1.73 m^2^) was associated with greater likelihood of error (IRR: 1.13; 95% CI 1.04, 1.24; *p* < 0.001 for FAB) or poorer performance (β: −3.66; 95% CI −5.64, −1.86; *p* < 0.001 for RBANS) following robust covariate adjustment (model 3). Full results for overall cognitive performance are provided in Table 2.

**TABLE 2:** Declining estimated Glomerular Filtration Rate (eGFR) and Overall Cognitive Function in Community‐Dwelling Older Adults

	Model 1		Model 2		Model 3	
Mini‐mental state (error)	IRR (95% CI)	*p*	IRR (95% CI)	*p*	IRR (95% CI)	*p*
eGFR_creat_						
>90 ml/min/1.73 m^2^	1.		1.		1.	
	(Ref.)		(Ref.)		(Ref.)	
75.0–89.9 ml/min/1.73 m^2^	1.30		1.10		1.11	
	(1.21, 1.38)	<0.001	(1.04, 1.19)	<0.001	(1.04, 1.20)	0.002
60.0–74.9 ml/min/1.73 m^2^	1.33		1.10		1.11	
	(1.24, 1.42)	<0.001	(1.03, 1.18)		(1.04, 1.19)	0.004
45.0–59.9 ml/min/1.73 m^2^	1.44		1.12		1.12	
	(1.35, 1.55)	<0.001	(1.05, 1.21)	<0.001	(1.04, 1.21)	0.004
<45 ml/min/1.73 m^2^	1.55		1.19		1.17	
	(1.43, 1.66)	<0.001	(1.10, 1.29)	<0.001	(1.08, 1.27)	<0.001

Frontal assessment battery (error)	IRR (95% CI)	*p*	IRR (95% CI)	*p*	IRR (95% CI)	*p*
eGFR_crea_						
>90 ml/min/1.73 m^2^	1.		1.		1.	
	(Ref.)		(Ref.)		(Ref.)	
75.0–89.9 ml/min/1.73 m^2^	1.23		1.05		1.04	
	(1.24, 1.33)	<0.001	(0.93, 1.14)	0.196	(0.97, 1.12)	0.246
60.0–74.9 ml/min/1.73 m^2^	1.33		1.05		1.04	
	(1.28, 1.37)	<0.001	(0.97, 1.13)	0.212	(0.96, 1.12)	0.322
45.0–59.9 ml/min/1.73 m^2^	1.44		1.07		1.04	
	(1.39, 1.50)	<0.001	(0.99, 1.15)	0.090	(0.97, 1.13)	0.272
<45 ml/min/1.73 m^2^	1.55		1.19		1.13	
	(1.58, 1.70)	<0.001	(1.09, 1.29)	<0.001	(1.04, 1.24)	0.004

RBANS total	β (95% CI)	*p*	β (95% CI)	*p*	β (95% CI)	*p*
eGFR_crea_						
>90 ml/min/1.73 m^2^	0		0		0	
	(Ref.)		(Ref.)		(Ref.)	
75.0–89.9 ml/min/1.73 m^2^	−2.11		−1.24		−0.68	0.373
	(−3.64, −0.59)	0.007	(−2.76, 0.28)	0.437	(−2.16, 0.81)	
60.0–74.9 ml/min/1.73 m^2^	−3.06		−1.24		−1.20	0.121
	(−4.61, −1.51)	<0.001	−1.24 (−2.76, 0.28)	0.032	(−2.72, 0.32)	
45.0–59.9 ml/min/1.73 m^2^	−4.72		−1.98		−1.46	0.091
	(−6.37, −3.06)	<0.001	(−3.66, −0.31)	0.020	(−3.14, 0.21)	
<45 ml/min/1.73 m^2^	−6.88		−4.87		−3.66	
	(−8.78, −5.99)	<0.001	(−6.82, −2.92)	<0.001	(−5.64, −1.86)	<0.001

*Note*: Model 1 refers to unadjusted associations. Model 2 adjusts for age, sex, body mass index and level of education. Model 3 adjusts for all covariates included in model 2 and additionally adjusts for systolic and diastolic blood pressure, total cholesterol:high density lipoprotein ratio, history of diabetes, history of cardiovascular and cerebrovascular disease, alcohol, smoking, polypharmacy (5 or more medications) and use of angiotensin‐converting enzyme inhibitors or angiotensin receptor blockers.

Abbreviations: eGFR, estimated glomerular filtration rate; RBANS, Repeatable Battery for Assessment of Neuropsychological Status; IRR, Incidence Rate Ratio; 95% CI, 95% Confidence Interval.

With regards to specific domains of neuropsychological performance, declining eGFR was associated with poorer performance on all RBANS domains with greater effect sizes seen for declining strata of eGFR. Following robust covariate adjustment, associations persisted for declining eGFR strata and poorer performance on the immediate memory domain (β: −5.13; 95% CI: −7.30, −2.95; *p* < 0.001 for eGFR <45 ml/min/1.73 m^2^). For visual‐spatial, language and attention domains, only eGFR <45 ml/min/1.73 m^2^ was significantly associated with poorer performance after robust covariate adjustment (β: −2.35; 95% CI: −4.71, −0.06; *p* = 0.049 for visual‐spatial; β: −1.71; 95% CI −3.38; −0.05, *p* = 0.044 for language; β: −2.96; 95% CI: −5.04, −0.88; *p* = 0.005 for attention). Finally, for delayed memory performance, declining eGFR strata was associated with poorer performance, in particular for eGFR <45 ml/min/1.73 m^2^ (β: −3.20; 95% CI: −5.50, −0.90; *p* = 0.006) See Table 3 for full results.

**TABLE 3:** Declining estimated Glomerular Filtration Rate (eGFR) and Domain‐Specific Neuropsychological Performance in Community‐Dwelling Older Adults

RBANS index I (immediate memory)	β (95% CI)	*p*	β (95% CI)	*p*	β (95% CI)	*p*
eGFR_creat_						
>90 ml/min/1.73 m^2^	0		0		0	
	(Ref.)		(Ref.)		(Ref.)	
75.0–89.9 ml/min/1.73 m^2^	−1.65 (−3.31, 0.01)	0.051	−2.46 (−4.10, −0.82)	0.003	−2.22 (−3.86, −0.58)	0.008
60.0–74.9 ml/min/1.73 m^2^	−2.64 (−4.33, −0.96)	0.002	−3.72 (−5.40, −2.06)	0.001	−3.34 (−5.02, −1.66)	<0.001
45.0–59.9 ml/min/1.73 m^2^	−3.80 (−5.60, −2.00)	<0.001	−4.60 (−6.42, −2.76)	<0.001	−3.70 (−5.56, −1.84)	<0.001
<45 ml/min/1.73 m^2^	−5.33 (−7.38, −3.29)	<0.001	−6.67 (−8.78, −4.53)	<0.001	−5.13 (−7.30, −2.95)	<0.001

RBANS index II (visual‐spatial)	β (95% CI)	*p*	β (95% CI)	*p*	β (95% CI)	*p*
eGFR_creat_						
>90 ml/min/1.73 m^2^	0		0		0	
	(Ref.)		(Ref.)		(Ref.)	
75.0–89.9 ml/min/1.73 m^2^	−2.51 (−4.33, −0.68)	0.007	−1.08 (−2.87, 0.71)	0.236	−1.12 (−2.90, 0.66)	0.884
60.0–74.9 ml/min/1.73 m^2^	−3.68 (−5.53, −1.83)	<0.001	−1.54 (−3.37, 0.29)	0.099	−1.46 (−3.29, 0.37)	0.962
45.0–59.9 ml/min/1.73 m^2^	−4.87 (−6.85, −2.89)	<0.001	−1.41 (−3.42, 0.59)	0.167	−0.84 (−2.85, 1.17)	0.610
<45 ml/min/1.73 m^2^	−7.72 (−9.98, −5.47)	<0.001	−3.91 (−6.21, −1.61)	0.001	−2.35 (−4.71, −0.06)	0.049

RBANS index III (language)	β (95% CI)	*p*	β (95% CI)	*p*	β (95% CI)	*p*
eGFR_creat_						
>90 ml/min/1.73 m^2^	0		0		0	
	(Ref.)		(Ref.)		(Ref.)	
75.0–89.9 ml/min/1.73 m^2^	−1.14 (−2.36, 0.07)	0.065	0.01 (−1.24, 1.27)	0.985	−0.17 (−1.37, 1.14)	0.855
60.0–74.9 ml/min/1.73 m^2^	−1.60 (−2.84, −0.37)	0.011	−0.35 (−1.62, 0.93)	0.592	−0.44 (−1.72, 0.84)	0.503
45.0–59.9 ml/min/1.73 m^2^	−2.33 (−3.65, −1.01)	0.001	−0.73 (−2.13, −0.67)	0.305	−0.68 (−2.10, 0.75)	0.350
<45 ml/min/1.73 m^2^	−3.06 (−4.56, −1.56)	<0.001	−2.07 (−3.69, −0.44)	0.013	−1.71 (−3.38, −0.05)	0.044

RBANS index IV (attention)	β (95% CI)	*p*	β (95% CI)	*p*	β (95% CI)	*p*
eGFR_creat_						
>90 ml/min/1.73 m^2^	0		0		0	
	(Ref.)		(Ref.)		(Ref.)	
75.0–89.9 ml/min/1.73 m^2^	−1.80 (−3.38, −0.21)	0.026	0.23 (−1.34, 1.80)	0.78	0.06 (−1.51, 1, 61)	0.949
60.0–74.9 ml/min/1.73 m^2^	−1.97 (−3.58, −0.36)	0.016	−0.63 (−2.22, 0.96)	0.535	−0.43 (−2.10, 1.10)	0.542
45.0–59.9 ml/min/1.73 m^2^	−4.18 (−5.90, −2.46)	<0.001	−0.80 (−2.56, 0.96)	0.375	−0.55 (−2.32, 1.22)	0.541
<45 ml/min/1.73 m^2^	−6.24 (−8.21, −4.27)	<0.001	−3.82 (−5.87, −1.78)	<0.001	−2.96 (−5.04, −0.88)	0.005

RBANS index V (delayed memory)	β (95% CI)	*p*	β (95% CI)	*p*	β (95% CI)	*p*
eGFR_creat_						
>90 ml/min/1.73 m^2^	0		0		0	
	(Ref.)		(Ref.)		(Ref.)	
75.0–89.9 ml/min/1.73 m^2^	−2.90 (−4.63, −1.18)	0.001	−1.75 (−3.49, −0.23)	0.047	−1.81 (−3.54, −0.07)	0.042
60.0–74.9 ml/min/1.73 m^2^	−3.23 (−4.98, −1.48)	<0.001	−2.10 (−3.86, −0.34)	0.019	−2.02 (−3.80, −0.24)	0.026
45.0–59.9 ml/min/1.73 m^2^	−4.72 (−6.59, −2.85)	<0.001	−2.71 (−4.65, −0.78)	0.006	−2.27 (−4.24, −0.31)	0.023
<45 ml/min/1.73 m^2^	−6.06 (−8.18, −3.94)	<0.001	−4.10 (−6.33, −1.85)	<0.001	−3.20 (−5.50, −0.90)	0.006

*Note*: Model 1 refers to unadjusted associations. Model 2 adjusts for age, sex, body mass index and level of education. Model 3 adjusts for all covariates included in model 2 and additionally adjusts for systolic and diastolic blood pressure, total cholesterol:high density lipoprotein ratio, history of diabetes, history of cardiovascular and cerebrovascular disease, alcohol, smoking, polypharmacy (5 or more medications) and use of angiotensin‐converting enzyme inhibitors or angiotensin receptor blockers.

Abbreviations: eGFR, estimated glomerular filtration rate; RBANS, Repeatable Battery for Assessment of Neuropsychological Status; IRR, Incidence Rate Ratio; 95% CI, 95% Confidence Interval.

As part of pre‐specified secondary analysis, we analysed the above models within three separate age strata: (i) age 60–70 years (n = 1757; 36.0%), (ii) age 70–80 years (n = 1881; 38.5%) and (iii) age >80 years (n = 1249; 25.6%). For the likelihood of error on the MMSE, associations were strongest for those aged <70 years, persisting for all eGFR strata after robust adjustment (IRR: 1.33; 95% CI 1.10, 1.62; *p* = 0.004 for eGFR <45 ml/min/1.73 m^2^ for instance). Whilst some associations for MMSE persisted with declining eGFR strata for those aged 70–80, associations were not observed for those aged >80 (Table S1). Associations between declining eGFR strata and likelihood of error on the FAB were more variable, with significant associations in those aged 60–70 years for eGFR 45–59.9 ml/min/1.73 m^2^ (IRR: 1.28; 95% CI 1.12, 1.48; *p* < 0.001) and eGFR 60–74.9 ml/min/1.73 m^2^ (IRR: 1.17; 95% CI: 1.05, 1.30; *p* = 0.003). Associations between declining eGFR and likelihood of error on the FAB were not seen for those aged >80. For total RBANS score, the greatest effect of declining eGFR was seen for those aged <70 years (β: −6.24; 95% CI: −10.44, −2.05; *p* = 0.004 fully adjusted for eGFR <45 ml/min/1.73 m^2^), whilst associations between declining eGFR and total RBANS were attenuated following adjustment for those aged 70–80 years, and not observed in those aged >80 years (Table 1).

These results were mirrored when analysing specific RBANS domains, with adults aged <70 years demonstrating the poorest performance with eGFR <45 ml/min/1.73 m^2^ in fully adjusted models in immediate memory (β: −7.79; 95% CI: −12.65, −2.93; *p* = 0.002), language (β: −4.10; 95% CI: −7.14, −1.06; *p* = 0.012) and attention (β: −6.83; 95% CI: −11.70, −1.96; *p* = 0.006). Whilst associations were seen in the 70–80 year old category for eGFR <45 ml/min/1.73 m^2^ and poorer performance for immediate memory (β: −4.92; 95% CI: −8.54, −1.29, *p* = 0.008) and attention (β: −5.59; 95% CI: −9.31, −1.87, *p* = 0.003), no associations on other domains were observed. Similarly, associations were seen in the oldest age strata (>80 years) for those with an eGFR <45 ml/min/1.73 m^2^ and poorer immediate memory performance (β: −17.76, 95% CI: −31.83, −3.68; *p* = 0.013 fully adjusted) whilst none were observed for other domains in those aged >80 years.

## 4 DISCUSSION

In the current study of nearly 5000 community‐dwelling older adults free from an established diagnosis of dementia, decreasing eGFR was found to be associated with a greater likelihood of error on tests of global cognitive function (MMSE) and executive function (FAB). Further, declining kidney function was associated with significantly poorer performance on neuropsychological tests of immediate memory, visuo‐spatial ability and attention. Of note, associations were greatest for those in the lowest strata of kidney function (eGFR <45 ml/min/1.73 m^2^) and for those aged 60–70 years, a group who may be particularly at risk and may benefit from potential preventative interventions.

The most striking findings from the current study involve the association between decreased kidney function and neuropsychological tests of immediate memory, attention and delayed memory. The strongest of these associations was seen for immediate memory, which has previously been reported to be affected, even in early‐stage CKD.^1^ The association between impaired attention and CKD has been known in the literature for some time and is supported by electrophysiological studies.^32^ Additionally, studies in patients undergoing haemodialysis have demonstrated reduced thickness and altered connectivity in the Pre‐Frontal Cortex (PFC), an area important for attention and inhibitory control.^33^, ^34^ In line with this evidence, a study comparing CKD‐associated cognitive impairment with Alzheimer Disease demonstrated more pronounced dysfunction in the frontal cortex in those with CKD.^35^ These converging lines of evidence, in the context of the current findings implicate an important role for immediate memory, attention and delayed with declining kidney function.

One of the interesting findings from our study are the results of the secondary analysis demonstrating the strongest associations for the youngest‐old (aged 60–70 years), but not necessarily for the oldest old. While the prevalence of reduced eGFR increases with age, we accounted for this by incorporating age as part of the CKD‐Epi formula in the first instance, but also in the use of age and as a covariate. Whilst eGFR is an important parameter, creatinine concentrations are age‐related and a consequence of analysing eGFR by creatinine in this age group is potential over diagnosis of CKD. Interestingly, previous reports have noted that in those above the age of 80 years, a significant decrease in eGFR measured using creatinine may be benign.^36^ This may explain the lack of association between reduced kidney function and cognitive performance in those aged >80 years in the current analysis, indicating that other ways of characterising the burden of CKD may be more useful in adults aged >80 years^37^ However, it is also worth considering that the lack of association in those aged >80 in the current study may reflect the accumulation of many other vascular and cognitive risk factors which are less important than age itself as a risk factor at older ages.^38^, ^39^

An important consideration in the current study is the renal biomarker that was used. We estimated eGFR from serum creatinine and applied the CKD‐Epi formula. This formula remains the most widely used estimate of kidney function in epidemiological studies in older adults. Despite this, previous studies using serum cystatin C to calculate eGFR have demonstrated associations with cystatin C and poorer cognitive function in older adults, and were not necessarily observed with eGFR estimates from creatinine alone, such as in the comprehensive Atherosclerosis Risk in Communities Study and the NICOLA study in Ireland.^40^, ^41^, ^42^, ^43^, ^44^ Further studies should include multiple kidney markers and assess their longitudinal impact on cognitive function, particularly in the domains of attention, visuo‐spatial function and immediate memory, in order to optimise cut‐points and select out individuals at greatest risk of further cognitive decline.

The aetiology of impaired cognitive function in CKD is becoming increasingly evident. Many of these focus on molecular mediators being retained in systemic circulation because of CKD.^1^ More recently, attention has directed towards the role of comorbid conditions (hypertension, cardiovascular disease), vascular dysfunction, inflammation, nutrition, anaemia and increased amyloid deposition.^1^ Additional mechanisms include endothelial dysfunction and sleep alterations in CKD leading to impaired glymphatic clearance and an important role for uraemic toxins and kidney neurotrophins such as Tumor Necrosis Factor and Neuropeptide Y (Neuropeptide Y).^44^, ^45^, ^46^, ^47^, ^48^ Further studies elucidating the direct and indirect mechanisms by which reduced kidney function may affect cognition are thus warranted.

An important consideration in the interpretation of the current findings is the relationship between CKD and vascular disease. Importantly, by classifying participants by eGFR criteria, we may have included patients with sub‐clinical vascular disease which of itself could negatively impact cognitive function. However, we were able to control for several important vascular risk factors (including smoking, hypertension, hypercholesterolaemia). It is possible there is an overlap between hypertension and CKD in the current study with the potential impact of CKD on cognition being mediated through blood pressure effects.^1^ Importantly though, we controlled for blood pressure levels at the time of patient recruitment as well as the use of antihypertensive medications. We were not however able to adjust for how long patients had their diagnosis of hypertension. It is also important to consider recent evidence in the use of Sodium Glucose Like Transporter inhibitors (SGLT2 inhibitors) in the treatment of proteinuric CKD.^49^ Further trials exploring the reno‐protective effects of these medications should assess for potential effects on cognitive outcomes.

An important limitation of the current study is its cross‐sectional nature, precluding any longitudinal analysis of the relationship between a decline in kidney function and decline in cognition in community dwelling older adults. Further, lack of Magnetic Resonance Imaging data to quantify vascular burden is an additional limitation and would have been a useful addition to the study. A final limitation of the current study is lack of data on whether those in the lowest strata of eGFR were on dialysis treatment. This may be a source of residual confounding given the presence of specific dialysis‐associated risk factors.^1^

There are several important implications from the current study. Our findings around the impact of CKD on cognitive function highlights the greatest effect sizes for immediate and delayed memory domains in addition to attention in individuals in the lowest strata of kidney function. These differences are likely to be clinically significant given their magnitude, although it is not possible from the current analysis to identify whether individuals met diagnostic criteria for mild cognitive impairment or dementia (notably, individuals had to be free from an established diagnosis of dementia to take part). The fact that these associations were strongest in those aged 60–70 demonstrates that potential multidomain preventative interventions (e.g FINGER) seeking to prevent cognitive decline in individuals with CKD may be best targeted at the youngest‐old with outcomes to include tests of immediate and delayed memory as well as attention. Future research is needed both to characterise these deficits longitudinally in individuals with CKD and examine if individuals with CKD benefit from targeted preventative interventions.

In conclusion, we assessed the relationship between kidney function and detailed cognitive and neuropsychological performance in nearly 5000 older adults. We demonstrated that reduced renal function was associated with significantly poorer performance on tests of immediate memory, visuo‐spatial function, and attention. These associations persisted following robust covariate adjustment and were particularly strong for the youngest‐old (<70 years), but less so in those aged 80 years or older. This study represents one of the largest of its kind to examine the relationship of eGFR and with neuropsychological performance, and the findings suggest that markers of early cognitive impairment in patients with reduced kidney function could be used to target preventative interventions to lower the risk of later cognitive decline.

## AUTHOR CONTRIBUTIONS

**Adam H. Dyer**, **Kevin McCarroll**, **Donal J. Sexton**: Study design; conception; interpretation. **Eamon Laird**, **Leane Hoey**, **Catherine F. Hughes**, **Helene McNulty**, **Mary Ward**, **J. J. Strain**, **Anne M. Molloy**, **Conal Cunningham**: Design; conception; conduct and oversight of the TUDA study. All authors read and approved the final manuscript prior to publication.

## CONFLICT OF INTEREST

The authors have no competing interests to declare.

## Supporting information

Supplementary Material S1

## Data Availability

The data that support the findings of this study are available from the corresponding author upon reasonable request.
