# Supplementary material for: Reduced kidney function is associated with poorer domain‐specific cognitive performance in community‐dwelling older adults
Source: Int J Geriatr Psychiatry. 2022 Jun 19;37(7):10.1002/gps.5771. doi: 10.1002/gps.5771 (PMC9327725; doi:10.1002/gps.5771)
Supplement: Supplementary file 1 — Supplementary Material S1 [file GPS-37-0-s001.docx]

|  | **Model 1** |  | **Model 2** |  | **Model 3** |  |
| --- | --- | --- | --- | --- | --- | --- |
| **Age <70 Years (n = 1,757)** |  |  |  |  |  |  |
| **Mini-Mental State (Error)** | **IRR (95% CI)** | **p** | **IRR (95% CI)** | **p** | **IRR (95% CI)** | **p** |
| eGFR_creat_  >90ml/min/1.73m^2^  75.0-89.9ml/min/1.73m^2^  60.0-74.9ml/min/1.73m^2^  45.0-59.9ml/min/1.73m^2^  <45ml/min/1.73m^2^ | 1. (Ref.)  1.13 (1.03, 1.24)  1.14 (1.03, 1.25)  1.28 (1.14, 1.43)  1.44 (1.19, 1.74) | 0.009  0.009  <0.001  <0.001 | 1. (Ref.)  1.09 (0.99, 1.19)  1.07 (0.98, 1.17)  1.14 (1.02, 1.27)  1.27 (1.04, 1.53) | 0.069  0.135  0.027  0.016 | 1. (Ref.)  1.10 (1.00, 1.20)  1.11 (1.01, 1.22)  1.17 (1.04, 1.32)  1.33 (1.10, 1.62) | 0.051  0.029  0.009  0.004 |
| **Frontal Assessment Battery (Error)** | **IRR (95% CI)** | **p** | **IRR (95% CI)** | **p** | **IRR (95% CI)** | **p** |
| eGFR_creat_  >90ml/min/1.73m^2^  75.0-89.9ml/min/1.73m^2^  60.0-74.9ml/min/1.73m^2^  45.0-59.9ml/min/1.73m^2^  <45ml/min/1.73m^2^ | 1. (Ref.)  1.12 (1.01, 1.23)  1.20 (1.08, 1.33)  1.39 (1.23, 1.57)  1.42 (1.16, 1.75) | 0.029  0.001  <0.001  0.001 | 1. (Ref.)  1.10 (0.99, 1.21)  1.16 (1.05, 1.27)  1.28 (1.13, 1.44)  1.29 (1.05, 1.58) | 0.079  0.004  <0.001  0.016 | 1. (Ref.)  1.09 (0.98, 1.20)  1.17 (1.05, 1.30)  1.28 (1.12, 1.48)  1.23 (0.99, 1.53) | 0.117  0.003  <0.001  0.057 |
| **RBANS Total** | **β (95% CI)** | **p** | **β (95% CI)** | **p** | **β (95% CI)** | **p** |
| eGFR_creat_  >90ml/min  75.0-89.9ml/min  60.0-74.9ml/min  45.0-59.9ml/min  <45ml/min | 0 (Ref.)  -0.51 (-2.38, 1.36)  -1.21 (-3.19, 0.77)  -4.07 (-6.56, -1.57)  -8.83 (-13.35, -4.31) | 0.595  0.230  0.001  <0.001 | 0 (Ref.)  -0.75 (-2.42, 0.93)  -0.95 (-2.67, 0.77)  -2.83 (-5.05, -0.61)  -7.99 (-12.13, -3.86) | 0.383  0.281  0.012  0.001 | 0 (Ref.)  -0.65 (-2.31, 1.01)  -0.91 (-2.64, 0.90)  -2.10 (-4.34, 0.15)  -6.24 (-10.44, -2.05) | 0.441  0.302  0.067  0.004 |
| **RBANS Index I**  **(Immediate Memory)** |  |  |  |  |  |  |
| eGFR_creat_  >90ml/min/1.73m^2^  75.0-89.9ml/min/1.73m^2^  60.0-74.9ml/min/1.73m^2^  45.0-59.9ml/min/1.73m^2^  <45ml/min/1.73m^2^ | 0 (Ref.)  -1.50 (-3.58, 0.57)  -1.24 (-3.44, 0.95)  -3.36 (-6.12, 0.60)  -8.62 (-13.59, -3.66) | 0.154  0.265  0.017  0.001 | 0 (Ref.)  -2.88 (-4.81, 0.93)  -2.68 (-4.69, -0.68)  -4.69 (-7.28, -2.11)  -9.96 (-14.71, -5.20) | 0.004  0.009  <0.001  <0.001 | 0 (Ref.)  -2.64 (-4.67, -0.70)  -2.38 (-4.40, -0.37)  -3.76 (-6.37, -1.14)  -7.79 (-12.65, -2.93) | 0.008  0.021  0.005  0.002 |
| **RBANS Index II**  **(Visual Spatial)** |  |  |  |  |  |  |
| eGFR_creat_  >90ml/min/1.73m^2^  75.0-89.9ml/min/1.73m^2^  60.0-74.9ml/min/1.73m^2^  45.0-59.9ml/min/1.73m^2^  <45ml/min/1.73m^2^ | 0 (Ref.)  -0.90 (-3.26, 1.45)  -1.97 (-4.46, 0.52)  -5.79 (-8.92, -2.65)  -9.47 (-15.17, -3.78) | 0.452  0.121  <0.001  0.001 | 0 (Ref.)  -0.03 (-2.19, 2.13)  -0.15 (-2.38, 2.07)  -3.30 (-4.63, -1.09)  -5.21 (-10.54, -0.11) | 0.978  0.892  0.224  0.055 | 0 (Ref.)  -0.68 (-2.89, 1.50)  -0.28 (-2.52, 1.96)  -1.22 (-4.11, 1.69)  -4.20 (-9.65, 1.24) | 0.950  0.808  0.411  0.130 |
| **RBANS Index III**  **(Language)** |  |  |  |  |  |  |
| eGFR_creat_  >90ml/min/1.73m^2^  75.0-89.9ml/min/1.73m^2^  60.0-74.9ml/min/1.73m^2^  45.0-59.9ml/min/1.73m^2^  <45ml/min/1.73m^2^ | 0 (Ref.)  -0.49 (-1.77, 0.80)  -1.13 (-2.49, 0.23)  -2.72 (-4.43, -1.01)  -4.90 (-7.99, -1.82) | 0.458  0.103  0.002  0.002 | 0 (Ref.)  -0.17 (-1.38, 1.04)  -0.48 (-1.73, 0.77)  -1.85 (-3.45, -0.24)  -4.44 (-7.41, -1.48) | 0.783  0.453  0.024  0.003 | 0 (Ref.)  -0.23 (-1.45, 0.99)  -0.61 (-1.87, 0.66)  -2.30 (-3.98, -0.62)  -4.10 (-7.14, -1.06) | 0.707  0.346  0.038  0.012 |
| **RBANS Index IV**  **(Attention)** |  |  |  |  |  |  |
| eGFR_creat_  >90ml/min/1.73m^2^  75.0-89.9ml/min/1.73m^2^  60.0-74.9ml/min/1.73m^2^  45.0-59.9ml/min/1.73m^2^  <45ml/min/1.73m^2^ | 0 (Ref.)  -0.15 (-2.23, 1.92)  -0.67 (-2.87, 1.52)  -3.45 (-6.21, -0.68)  -9.81 (-14.78, -4.84) | 0.885  0.548  0.015  <0.001 | 0 (Ref.)  0.47 (-1.47, 2.42)  0.96 (-1.04, 2.96)  -0.71 (-3.28, 1.86)  -8.60 (-13.07, -3.59) | 0.633  0.348  0.590  <0.001 | 0 (Ref.)  0.53 (-1.41, 2.48)  0.75 (-1.27, 2.77)  -0.37 (-2.99, -2.26)  -6.83 (-11.70, -1.96) | 0.592  0.466  0.783  0.006 |
| **RBANS Index V**  **(Delayed Memory)** |  |  |  |  |  |  |
| eGFR_creat_  >90ml/min/1.73m^2^  75.0-89.9ml/min/1.73m^2^  60.0-74.9ml/min/1.73m^2^  45.0-59.9ml/min/1.73m^2^  <45ml/min/1.73m^2^ | 0 (Ref.)  -0.42 (-2.34, 1.50)  -1.01 (-3.05, 1.01)  -1.96 (-4.51, 0.60)  -4.27 (-8.86, 0.32) | 0.669  0.327  0.133  0.068 | 0 (Ref.)  -1.09 (-2.91, 0.72)  -1.76 (-3.64, 0.11)  -2.38 (-4.78, 0.03)  -4.67 (-9.12, -0.22) | 0.237  0.066  0.053  0.039 | 0 (Ref.)  -1.07 (-2.90, 0.75)  -1.65 (-2.69, 0.25)  -1.70 (-4.17, 0.75)  -2.95 (-7.53, 1.61) | 0.248  0.089  0.174  0.205 |
| **Age 70-80 Years (n = 1,881)** |  |  |  |  |  |  |
| **Mini-Mental State (Error)** | **IRR (95% CI)** | **p** | **IRR (95% CI)** | **p** | **IRR (95% CI)** | **p** |
| eGFR_creat_  >90ml/min/1.73m^2^  75.0-89.9ml/min/1.73m^2^  60.0-74.9ml/min/1.73m^2^  45.0-59.9ml/min/1.73m^2^  <45ml/min/1.73m^2^ | 1 (Ref.)  1.16 (1.03, 1.32)  1.15 (1.02, 1.31)  1.22 (1.07, 1.40)  1.25 (1.09, 1.44) | 0.018  0.028  0.002  0.002 | 1 (Ref.)  1.05 (0.96, 1.15)  1.09 (0.99, 1.20)  1.12 (1.01, 1.25)  1.26 (1.10, 1.45) | 0.305  0.080  0.031  0.001 | 1 (Ref.)  1.06 (0.96, 1.17)  1.11 (1.00, 1.22)  1.12 (1.00, 1.25)  1.23 (1.07, 1.42) | 0.221  0.043  0.049  0.005 |
| **Frontal Assessment Battery (Error)** | **IRR (95% CI)** | **p** | **IRR (95% CI)** | **p** | **IRR (95% CI)** | **p** |
| eGFR_creat_  >90ml/min/1.73m^2^  75.0-89.9ml/min/1.73m^2^  60.0-74.9ml/min/1.73m^2^  45.0-59.9ml/min/1.73m^2^  <45ml/min/1.73m^2^ | 1 (Ref.)  0.91 (0.81, 1.03)  0.89 (0.79, 1.00)  0.91 (0.80, 1.03)  1.10 (0.96, 1.26) | 0.131  0.052  0.129  0.178 | 1 (Ref.)  1.05 (0.95, 1.16)  1.06 (0.96, 1.18)  1.14 (1.02, 1.28)  1.28 (0.11, 1.48) | 0.338  0.256  0.021  0.001 | 1 (Ref.)  1.05 (0.95, 1.16)  1.05 (0.94, 1.17)  1.10 (0.99, 1.24)  1.20 (0.04, 1.40) | 0.336  0.325  0.084  0.014 |
| **RBANS Total** | **β (95% CI)** | **p** | **β (95% CI)** | **p** | **β (95% CI)** | **p** |
| eGFR_creat_  >90ml/min/1.73m^2^  75.0-89.9ml/min/1.73m^2^  60.0-74.9ml/min/1.73m^2^  45.0-59.9ml/min/1.73m^2^  <45ml/min/1.73m^2^ | 0 (Ref.)  -0.82 (-3.96, 2.33)  -1.07 (-4.25, 2.11)  -3.51 (-6.83, -0.21)  -5.24 (-8.92, -1.58) | 0.612  0.510  0.037  0.005 | 0 (Ref.)  0.51 (-1.49, 2.50)  0.41 (-1.65, 2.48)  -3.37 (-5.75, -1.02)  -4.43 (-7.68, -1.18) | 0.619  0.697  0.005  0.008 | 0 (Ref.)  0.15 (-1.83, 2.13)  -0.15 (-1.91, 2.21)  -3.07 (-5.41, -0.72)  -3.29 (-6.56, -0.02) | 0.883  0.888  0.010  0.049 |
| **RBANS Index I**  **(Immediate Memory)** |  |  |  |  |  |  |
| eGFR_creat_  >90ml/min/1.73m^2^  75.0-89.9ml/min/1.73m^2^  60.0-74.9ml/min/1.73m^2^  45.0-59.9ml/min/1.73m^2^  <45ml/min/1.73m^2^ | 0 (Ref.)  -2.10 (-5.42, 1.2)  -3.10 (-6.45, 0.26)  -5.35 (-8.84, -1.86)  -6.85 (-10.72, -2.99) | 0.215  0.070  0.003  0.001 | 0 (Ref.)  -2.09 (-4.29, 0.12)  -1.98 (-4.26, 0.31)  -4.82 (-7.41, -2.23)  -6.56 (-10.12, -3.01) | 0.064  0.090  <0.001  <0.001 | 0 (Ref.)  -2.14 (-4.34, 0.06)  -1.88 (-4.18, 0.41)  -4.18 (-6.78, -1.57)  -4.92 (-8.54, -1.29) | 0.057  0.107  0.002  0.008 |
| **RBANS Index II**  **(Visual Spatial)** |  |  |  |  |  |  |
| eGFR_creat_  >90ml/min/1.73m^2^  75.0-89.9ml/min/1.73m^2^  60.0-74.9ml/min/1.73m^2^  45.0-59.9ml/min/1.73m^2^  <45ml/min/1.73m^2^ | 0 (Ref.)  -0.13 (-3.81, 3.55)  -0.66 (-4.37, 3.06)  -1.48 (-5.34, 2.38)  -2.89 (-7.16, 1.40) | 0.945  0.729  0.452  0.186 | 0 (Ref.)  0.75 (-1.73, 2.32)  0.38 (-2.18, 2.95)  -1.74 (-4.66, 1.16)  -2.34 (-6.36, 1.69) | 0.554  0.770  0.238  0.254 | 0 (Ref.)  0.20 (-2.25, 2.66)  -0.28 (-2.84, 2.28)  -1.82 (-4.73, 1.10)  -1.81 (-5.88, 2.26) | 0.870  0.832  0.220  0.383 |
| **RBANS Index III**  **(Language)** |  |  |  |  |  |  |
| eGFR_creat_  >90ml/min/1.73m^2^  75.0-89.9ml/min/1.73m^2^  60.0-74.9ml/min/1.73m^2^  45.0-59.9ml/min/1.73m^2^  <45ml/min/1.73m^2^ | 0 (Ref.)  0.45 (-1.93, 2.82)  0.96 (-1.44, 3.36)  -0.41 (-1.44, 3.36)  -1.61 (-4.37, 1.15) | 0.713  0.433  0.748  0.253 | 0 (Ref.)  0.73 (-0.73, 2.19)  1.40 (-0.12, 2.91)  -1.15 (-2.87, 0.56)  -2.91 (-5.26, -0.55) | 0.325  0.070  0.187  0.016 | 0 (Ref.)  0.55 (-0.91, 2.01)  1.30 (-0.23, 2.81)  -1.09 (-2.82, 0.65)  -2.59 (-4.63, 0.18) | 0.460  0.095  0.217  0.069 |
| **RBANS Index IV**  **(Attention)** |  |  |  |  |  |  |
| eGFR_creat_  >90ml/min/1.73m^2^  75.0-89.9ml/min/1.73m^2^  60.0-74.9ml/min/1.73m^2^  45.0-59.9ml/min/1.73m^2^  <45ml/min/1.73m^2^ | 0 (Ref.)  0.16 (-3.13, 3.45)  0.48 (-2.84, 3.80)  -2.17 (-5.62, 1.28)  -5.25 (-9.08, -1.41) | 0.924  0.779  0.218  0.007 | 0 (Ref.)  1.37 (-0.90, 3.64)  1.82 (-0.53, 4.16)  -2.65 (-5.31, -0.00)  -6.09 (-9.76, -2.42) | 0.236  0.128  0.050  0.001 | 0 (Ref.)  0.80 (-1.45, 3.06)  1.24 (-1.10, 3.58)  -2.76 (-5.43, -0.90)  -5.59 (-9.31, -1.87) | 0.460  0.095  0.043  0.003 |
| **RBANS Index V**  **(Delayed Memory)** |  |  |  |  |  |  |
| eGFR_creat_  >90ml/min/1.73m^2^  75.0-89.9ml/min/1.73m^2^  60.0-74.9ml/min/1.73m^2^  45.0-59.9ml/min/1.73m^2^  <45ml/min/1.73m^2^ | 0 (Ref.)  -3.09 (-6.73, 0.55)  -2.93 (-6.61, 0.75)  -4.99 (-8.82, -1.16)  -5.30 (-9.54, -1.06) | 0.096  0.118  0.011  0.014 | 0 (Ref.)  -0.85 (-3.11, 1.42)  -1.28 (-3.63, 1.06)  -3.69 (-6.34, -1.03)  -1.98 (-5.63, 1.67) | 0.463  0.283  0.007  0.288 | 0 (Ref.)  -1.00 (-3.26, 1.27)  -1.27 (-3.63, 1.09)  -3.29 (-5.97, -0.60)  -0.85 (-4.57, 2.88) | 0.388  0.290  0.016  0.656 |
| **Age >80 Years (n = 1,249)** |  |  |  |  |  |  |
| **Mini-Mental State (Error)** | **IRR (95% CI)** | **p** | **IRR (95% CI)** | **p** | **IRR (95% CI)** | **p** |
| eGFR_creat_  >90ml/min/1.73m^2^  75.0-89.9ml/min/1.73m^2^  60.0-74.9ml/min/1.73m^2^  45.0-59.9ml/min/1.73m^2^  <45ml/min/1.73m^2^ | 1.15 (0.75, 1.75)  1.17 (0.76, 1.78)  1.17 (0.77, 1.78)  1.24 (0.81, 1.89) | 0.525  0.477  0.465  0.315 | 1 (Ref.)  1.21 (0.79, 1.85)  1.22 (0.80, 1.86)  1.20 (0.78, 1.83)  1.26 (0.83, 1.93) | 0.378  0.366  0.405  0.281 | 1 (Ref.)  1.19 (0.78, 1.82)  1.19 (0.78, 1.82)  1.18 (0.77, 1.80)  1.24 (0.81, 1.90) | 0.423  0.420  0.455  0.323 |
| **Frontal Assessment Battery (Error)** | **IRR (95% CI)** | **p** | **IRR (95% CI)** | **p** | **IRR (95% CI)** | **p** |
| eGFR_creat_  >90ml/min/1.73m^2^  75.0-89.9ml/min/1.73m^2^  60.0-74.9ml/min/1.73m^2^  45.0-59.9ml/min/1.73m^2^  <45ml/min/1.73m^2^ | 1 (Ref.)  1.27 (0.81, 2.00)  1.26 (0.80, 1.98)  1.30 (0.83, 2.04)  1.40 (0.89, 2.20) | 0.299  0.319  0.259  0.148 | 1 (Ref.)  1.27 (0.81, 2.01)  1.23 (0.78, 1.94)  1.22 (0.77, 1.92)  1.30 (0.82, 2.04) | 0.297  0.376  0.395  0.263 | 1 (Ref.)  1.24 (0.79, 1.96)  1.19 (0.76, 1.88)  1.19 (0.75, 1.87)  1.26 (0.80, 1.99) | 0.354  0.452  0.464  0.322 |
| **RBANS Total** |  |  | **β (95% CI)** | **p** | **β (95% CI)** | **p** |
| eGFR_creat_  >90ml/min/1.73m^2^  75.0-89.9ml/min/1.73m^2^  60.0-74.9ml/min/1.73m^2^  45.0-59.9ml/min/1.73m^2^  <45ml/min/1.73m^2^ | 0 (Ref.)  -7.67 (-20.72, 5.38)  -9.59 (-22.61, 3.43)  -8.18 (-21.19, 4.82)  -10.80 (-23.81, 2.22) | 0.250  0.149  0.218  0.104 | 0 (Ref.)  -9.49 (-21.84, 2.86)  -11.15 (-23.48, 1.19)  -8.96 (-21.30, 3.38)  -11.65 (-24.00, 0.71) | 0.132  0.076  0.155  0.065 | 0 (Ref.)  -9.13 (-21.51, 3.24)  -10.78 (-23.14, 1.58)  -8.53 (-20.91, 3.84)  -11.13 (-23.54, 1.27) | 0.148  0.087  0.177  0.079 |
| **RBANS Index I**  **(Immediate Memory)** |  |  |  |  |  |  |
| eGFR_creat_  >90ml/min/1.73m^2^  75.0-89.9ml/min/1.73m^2^  60.0-74.9ml/min/1.73m^2^  45.0-59.9ml/min/1.73m^2^  <45ml/min/1.73m^2^ | 0 (Ref.)  -13.60 (-28.19, 0.99)  -16.68 (-31.24, -2.13)  -15.30 (-29.83, -0.76)  -16.96 (-31.51, -2.41) | 0.068  0.025  0.039  0.022 | 0 (Ref.)  -15.65 (-29.67, -1.63)  -18.79 (-32.80, -4.79)  -16.85 (-30.86, -2.84)  -18.40 (-32.43, -4.37) | 0.029  0.009  0.018  0.010 | 0 (Ref.)  -15.25 (-29.30, -1.20)  -18.32 (-32.47, -4.40)  -16.30 (-30.34, -2.25)  -17.76 (-31.83, -3.68) | 0.033  0.010  0.023  0.013 |
| **RBANS Index II**  **(Visual Spatial)** |  |  |  |  |  |  |
| eGFR_creat_  >90ml/min/1.73m^2^  75.0-89.9ml/min/1.73m^2^  60.0-74.9ml/min/1.73m^2^  45.0-59.9ml/min/1.73m^2^  <45ml/min/1.73m^2^ | 0 (Ref.)  2.60 (-12.50, 17.69)  1.10 (-13.95, 16.15)  2.83 (-12.21, 17.87)  -1.41 (-16.47, 13.63) | 0.736  0.886  0.712  0.852 | 0 (Ref.)  2.11 (-11.93, 16.16)  1.69 (-12.34, 15.72)  4.20 (-9.83, 18.35)  -0.19 (-14.24, 13.87) | 0.768  0.813  0.557  0.979 | 0 (Ref.)  2.06 (-11.96, 16.09)  1.85 (-12.16, 15.86)  4.43 (-9.59, 18.45)  0.27 (-13.78, 14.33) | 0.773  0.796  0.536  0.969 |
| **RBANS Index III**  **(Language)** |  |  |  |  |  |  |
| eGFR_creat_  >90ml/min/1.73m^2^  75.0-89.9ml/min/1.73m^2^  60.0-74.9ml/min/1.73m^2^  45.0-59.9ml/min/1.73m^2^  <45ml/min/1.73m^2^ | 0 (Ref.)  -1.80 (-14.27, 10.67)  -3.41 (-15.86, 9.03)  -2.07 (-14.50, 10.36)  -2.66 (-15.10, 9.77) | 0.777  0.591  0.745  0.675 | 0 (Ref.)  -2.87 (-15.20, 9.46)  -4.45 (-16.77, 7.86)  -2.72 (-15.04, 9.60)  -3.13 (-15.47, 9.20) | 0.648  0.479  0.665  0.618 | 0 (Ref.)  -2.95 (-15.30, 9.40)  -4.60 (-16.94, 7.74)  -2.96 (-15.32, 9.39)  -3.45 (-15.83, 8.93) | 0.640  0.465  0.638  0.585 |
| **RBANS Index IV**  **(Attention)** |  |  |  |  |  |  |
| eGFR_creat_  >90ml/min/1.73m^2^  75.0-89.9ml/min/1.73m^2^  60.0-74.9ml/min/1.73m^2^  45.0-59.9ml/min/1.73m^2^  <45ml/min/1.73m^2^ | 0 (Ref.)  -7.84 (-20.00, 4.32)  -7.94 (-20.07, 4.19)  -8.58 (-20.70, 3.54)  -9.90 (-22.02, 2.23) | 0.206  0.200  0.165  0.110 | 0 (Ref.)  -8.75 (-20.30, 2.81)  -8.50 (-20.15, 2.94)  -8.57 (-20.12, 2.98)  -10.06 (-21.63, 1.51) | 0.138  0.144  0.146  0.088 | 0 (Ref.)  -7.49 (-18.99, 4.01)  -7.32 (-18.81, 4.16)  -7.24 (-18.74, 4.26)  -8.54 (-20.06, 2.99) | 0.202  0.211  0.217  0.147 |
| **RBANS Index V**  **(Dleayed Memory)** |  |  |  |  |  |  |
| eGFR_creat_  >90ml/min/1.73m^2^  75.0-89.9ml/min/1.73m^2^  60.0-74.9ml/min/1.73m^2^  45.0-59.9ml/min/1.73m^2^  <45ml/min/1.73m^2^ | 0 (Ref.)  -9.95 (-25.55, 5.64)  -9.46 (-25.01, 6.09)  -9.15 (-24.69, 6.39)  -10.61 (-26.15, 4.94) | 0.211  0.233  0.248  0.181 | 0 (Ref.)  -12.40 (-27.66, 2.88)  -11.90 (-27.16, 3.35)  -11.18 (-26.44, 4.08)  -12.55 (-27.83, 2.73) | 0.112  0.126  0.151  0.107 | 0 (Ref.)  -12.08 (-27.40, 3.42)  -11.56 (-26.87, 3.24)  -11.56 (-26.87, 3.75)  -12.50 (-27.85, 2.85) | 0.122  0.139  0.161  0.111 |

*Note*: Model 1 refers to unadjusted associations. Model 2 adjusts for age, sex, body mass index and level of education. Model 3 adjusts for all covariates included in model 2 and additionally adjusts for systolic and diastolic blood pressure, total cholesterol:high density lipoprotein ratio, history of diabetes, history of cardiovascular and cerebrovascular disease, alcohol, smoking, polypharmacy (5 or more medications) and use of angiotensin-converting enzyme inhibitors or angiotensin receptor blockers. Abbreviations: eGFR: estimated glomerular filtration rate. RBANS: Repeatable Battery for Assessment of Neuropsychological Status; IRR: Incidence Rate Ratio; 95% CI: 95% Confidence Interval.
